# Review of the Genus *Dolophrades* Bates, 1884 (Coleoptera: Cerambycidae: Lamiinae: Morimopsini), with Description of a New Species from China

**DOI:** 10.3390/insects17101031

**Published:** 2026-10-08

**Authors:** Chaokun Yang, Zixiang Wang, Peng Zhan, Ping Wang, Guanglin Xie, Wenkai Wang, Hong Liu

**Affiliations:** 1 Institute of Entomology, College of Agriculture, Yangtze University, Jingzhou 434025, China; yang1764847081@163.com (C.Y.); fxver9@outlook.com (Z.W.); wangping1992@yangtzeu.edu.cn (P.W.); wwk@yangtzeu.edu.cn (W.W.); 2 Hubei Provincial Key Laboratory for Protection and Application of Special Plant Germplasm in Wuling Area of China, College of Life Sciences, South-Central Minzu University, Wuhan 430074, China; 2026010093@mail.scuec.edu.cn; 3 MARA Key Laboratory of Sustainable Crop Production in the Middle Reaches of the Yangtze River (Co-Construction by Ministry and Province), College of Agriculture, Yangtze University, Jingzhou 434025, China

**Keywords:** identification key, longhorned beetle, morphology, nomenclature, synonym, taxonomy

## Abstract

Taxonomic notes on the genus *Dolophrades* Bates, 1884 are presented. *D. tianmushanensis* Yang, Xie & W. Wang sp. nov. from Tianmushan National Nature Reserve, Zhejiang Province, China, is described and illustrated. *D. punctatus* (Pic, 1934), stat. restit., and *D. subdenudatus* Gressitt, 1938, stat. restit., are reinstated as separate valid species. The taxon originally described as *Cereopsius decoloratus* var. *annulicornis* Schwarzer, 1925, subsequently treated as *D. annulicornis* (Schwarzer, 1925), is restored to subspecific rank as *Peblephaeus decoloratus annulicornis* (Schwarzer, 1925), stat. restit. Six species of the genus are recognized in this treatment. A key to the known species of the genus and a distribution map are provided.

## Simple Summary

*Dolophrades* Bates, 1884 is a small genus of Asian longhorn beetles whose species boundaries have remained unclear because several taxa were historically confused or treated as the same species. By examining specimens, photographs of type material, and original descriptions, we reassessed the taxonomy of the genus and clarified the identities of several previously confused taxa. We also describe a new species from Tianmushan National Nature Reserve, Zhejiang Province, China. Six species are recognized in *Dolophrades*, and updated illustrations, an identification key, and a distribution map are provided.

## 1. Introduction

Bates (1884) established the genus *Dolophrades* for *Dolophrades terrenus* Bates, 1884 from Hitoyoshi, Japan [1]. Subsequently, Schwarzer (1925) described *Cereopsius decolorata* var. *annulicorne* from Pilam, Taiwan, China [2], which is currently treated as *Dolophrades annulicornis* (Schwarzer, 1925). Pic (1934) established the genus *Dorcadiohammus* for *Dorcadiohammus punctatus* from Ile Formose (present-day Taiwan, China) [3], and Gressitt (1938) described *Dolophrades subdenudatus* from central and southwestern Formosa (present-day Taiwan, China) [4]. Breuning (1950) synonymized *Dorcadiohammus* under *Dolophrades*, and treated *Dorcadiohammus punctatus* and *Dolophrades subdenudatus* as junior synonyms of *D*. *annulicornis* [5]. Later, Breuning (1958) described *D. birmanus* from Ruby Mines (Mogok), Myanmar [6], and Holzschuh (2003) described *D. mustanganus* from Dhawalagiri, Kali-Gandaki-Khola, Kalopani, Nepal [7]. Currently, four species are recognized in *Dolophrades*, distributed in East, Southeast, and South Asia [8,9].

In this paper, we review *Dolophrades*, describe a new species from Zhejiang Province, and reinstate *D. punctatus* (Pic, 1934) and *D. subdenudatus* Gressitt, 1938 as separate valid species. The taxon originally described as *Cereopsius decoloratus* var. *annulicornis* Schwarzer, 1925 is excluded from *Dolophrades* and referred to *Peblephaeus* Kusama & Takakuwa, 1984. Photographs of the examined types of *Dorcadiohammus punctatus* Pic, 1934 (original combination), *Dolophrades annulicornis* (Schwarzer, 1925), *D. birmanus* Breuning, 1958, and *D. terrenus* Bates, 1884, the allotypes of *D. subdenudatus* Gressitt, 1938 and type-series material of *Cereopsius decolorata* Schwarzer, 1925 and the original type series of *Cereopsius decolorata* var. *annulicorne* Schwarzer, 1925 are provided, and a key to the species of the genus *Dolophrades* Bates is given.

## 2. Materials and Methods

Specimens from the following institutional collections were examined for this study:

**CAS**, California Academy of Sciences, San Francisco, CA, USA.

**ICYZU**, Institute Collection, Yangtze University, Jingzhou, Hubei, China.

**IRSNB**, Institut royal des Sciences naturelles de Belgique, Bruxelles, Belgium.

**MNHN**, Muséum national d’Histoire naturelle, Paris, France.

**NHMUK**, Natural History Museum, London, UK.

**SMF**, Senckenberg Forschungsinstitut und Naturmuseum, Frankfurt am Main, Germany.

**USNM (=NMNH)**, National Museum of Natural History (Smithsonian) [formerly, United States National Museum], Washington, DC, USA.

The overall dimensions of the holotypes *D. punctatus* Pic, 1934 and *D. birmanus* Breuning, 1958; the allotypes’ body length and humeral width of elytra of *Dolophrades subdenudatus* Gressitt, 1938 were obtained based on the scale bars contained in the photographs. The dimensional data of the holotype *D. terrenus* Bates, 1884 were obtained based on the original description.

The male terminalia were prepared by first soaking the whole beetle in hot water for several minutes, then opening the abdomen from the abdominal apex along the dorsopleural margin. The terminalia were then removed with fine forceps and ophthalmic scissors, and later cleared in 10% NaOH solution (Guangdong Xinchengyuan Technology Co., Ltd., Huizhou, Guangdong, China) at 80–100 °C for several minutes.

Photographs of *Dolophrades tianmushanensis* Yang, Xie & W. Wang sp. nov. were taken with a Canon EOS 5D Mark II digital camera (Canon Inc., Tokyo, Japan) equipped with a LAOWA 100 mm F/2.8 2× Ultra Macro APO lens (Anhui Changgeng Optics Technology Co., Ltd., Hefei, China) in combination with Helicon Remote software (ver. 4.5.9). Detailed images of the male terminalia and morphological structures of the new species were taken with a LAOWA 25 mm F/2.8 2.5–5× Ultra Macro lens (Anhui Changgeng Optics Technology Co., Ltd., Hefei, China) using the same software. Photographs of type specimens of previously described species were provided by the respective institutional collections or their staff, as acknowledged below, and were not taken using the photographic equipment described above. The distribution map was prepared using QGIS 3.38.3 based on locality data from examined specimens, original descriptions, and published records. All figures were assembled and edited using Adobe Photoshop 2025, version 26.7.0.15; photographs supplied by institutional collections were otherwise retained without alteration to morphological details.

## 3. Results

***Dolophrades*** **Bates, 1884**

Chinese common name: 朵天牛属

*Dolophrades* Bates, 1884: 237 [1]. Type species: *Dolophrades terrenus* Bates, 1884, by monotypy.

*Dorcadiohammus* Pic, 1934: 8 [3]. Type species: *Dorcadiohammus punctatus* Pic, 1934, by monotypy.

**Redescription.** Body small, narrowly elongate-ovate, densely covered with short pubescence; head, pronotum, and elytra densely punctate. Vertex entire and slightly concave; frons subquadrate, slightly narrower at lower margin. Eyes coarsely faceted, inner margin shallowly emarginate; lower lobes small, longer than wide. Palpi with apical segments ovate, shortly truncate. Antennal tubercles distinctly raised, widely separated. Antennae longer than half body length, without cilia beneath; scape subcylindrical, with an open apical cicatrix bordered by a small carina; antennomere 3 more than 2 times as long as scape; antennomere 4 longer than scape. Pronotum quadrate, longer than wide, with small lateral tubercles. Scutellum tongue-shaped, apex rounded. Elytra elongate-oval, slightly wider than widest part of pronotum, gradually narrowed posteriorly from middle, humeri slightly produced; apices rounded or truncate; disc slightly convex, gradually sloping posteriorly, coarsely and sublineately punctate. Hind wings absent. Prosternum and mesosternum ventrally convex; metasternum slightly shortened. Prosternal process narrow, lower than procoxae, arched; mesosternal process broad, its ventral surface evenly declivous posteriorly, impunctate; procoxal cavities slightly open posteriorly; mesocoxal cavities open laterally. Legs long; femora claviform, mesotibiae with an oblique groove near outer apex. Claws simple, widely divergent.

**Figure 1:**
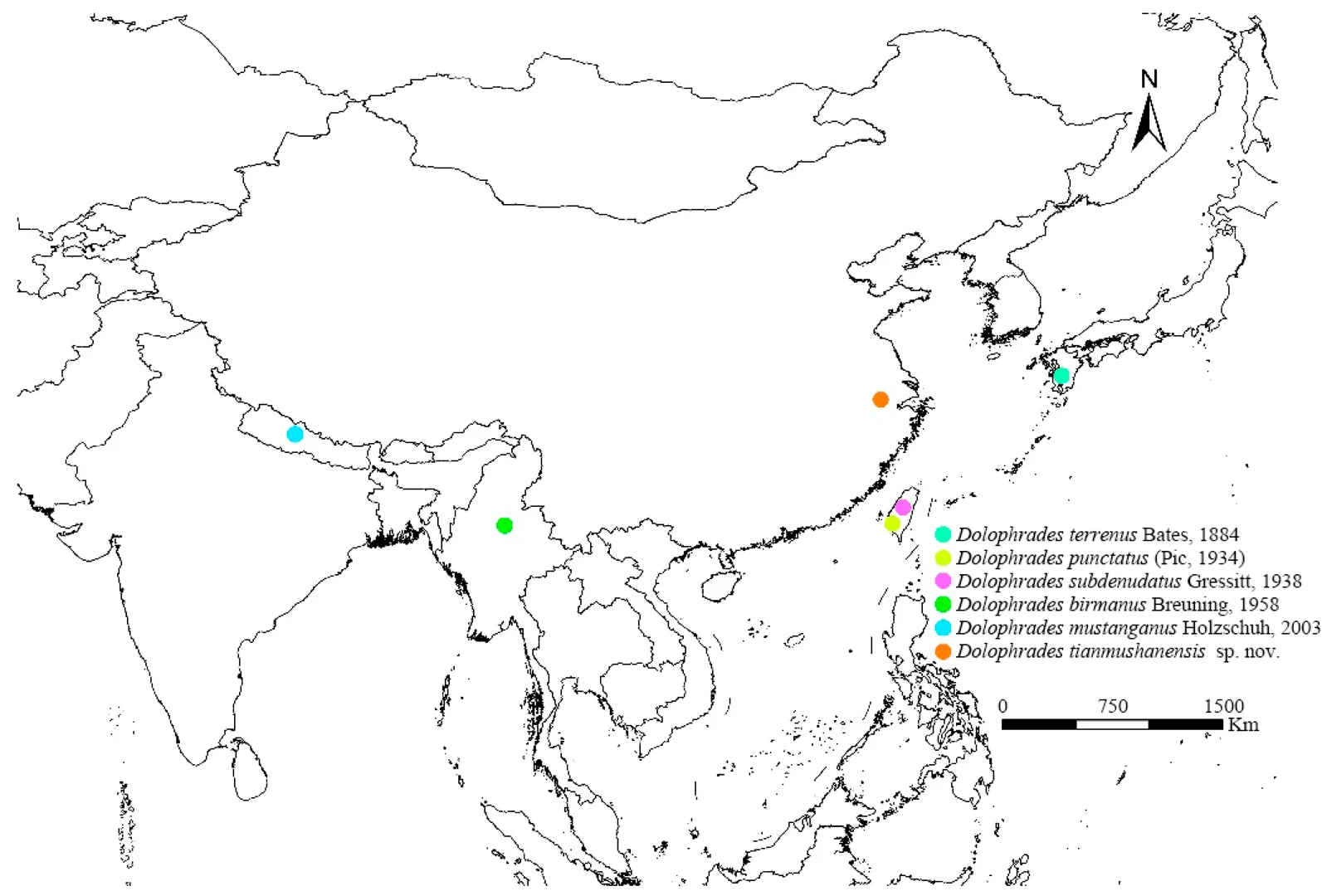
Distribution map of species of the genus *Dolophrades* Bates, 1884.

**Distribution.** China, Japan, Myanmar, Nepal. (Figure 1).

***Dolophrades terrenus* Bates, 1884**

Chinese common name: 朵天牛

Figure 2

*Dolophrades terrenus* Bates, 1884: 238 [1], pl. I, fig. 10. Type locality: Japan (Hitoyoshi); Aurivillius 1922: 65 (catalogue) [10]; Breuning 1961: 309 (catalogue) [11]; Hua 2002: 204 (catalogue) [12]; Hasegawa 2007: 569 (faune) [13]; Löbl & Smetana 2010: 289 (catalogue) [14]; Lin & Tavakilian 2019: 339 (catalogue) [8]; Yamasako 2020: 30 (distribution) [15].

**Redescription.** Male. Body length 10 mm, humeral width 2.7 mm.

Body yellowish-brown, densely covered with yellowish-brown pubescence. Head densely punctate; frons wider than high, with a fine median groove not extending to occiput. Antennal tubercles prominent, widely separated from each other. Antennae cylindrical; scape with cicatrix at apex; bases of antennomeres 3–11 with greyish-yellow pubescence. Pronotum longer than wide; lateral tubercle large, acute; disc uneven, densely punctate except below lateral tubercles. Scutellum thickly covered with golden pubescence. Elytra elongate-oval, coarsely punctate, punctures partially in rows, becoming sparser at posterior middle. Prosternum sparsely punctured medially; prosternal process almost smooth, narrow, lower than coxae, gradually widening at apex; procoxal cavities slightly open posteriorly. Mesosternal process broad, slightly convex at apex; mesocoxal cavities open externally to mesepimera; mesepisternum without punctures. Legs moderately long, femora fusiform, mesotibia with oblique groove near outer apex. Metafemur not reaching apex of abdominal ventrite V. Metatarsomere I longest, not longer than II+III combined.

**Figure 2:**
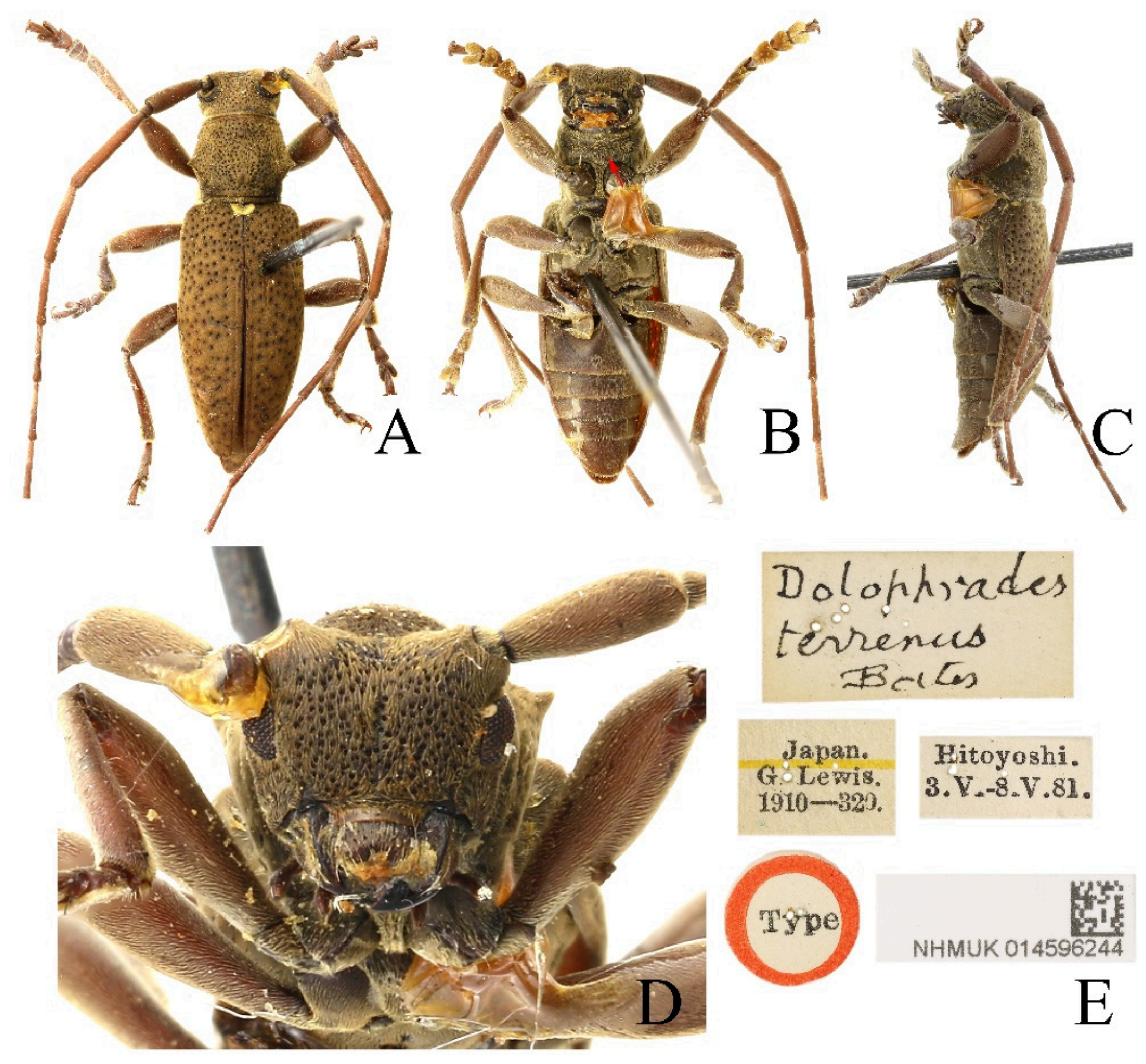
Holotype of *Dolophrades terrenus* Bates, 1884. (**A**) Dorsal view; (**B**) ventral view, red arrows indicate the shallow coarse punctures on the prosternum; (**C**) lateral view; (**D**) frontal view; (**E**) labels.

**Type material examined.** Holotype of *Dolophrades terrenus*, male (NHMUK); label details are shown in Figure 2E.

**Distribution.** Japan (Hitoyoshi).

**Comments.** Bates (1884) described *Dolophrades terrenus* based on a single male specimen from Hitoyoshi, Japan. Examination of the type specimen deposited in the Natural History Museum, London (NHMUK) (formerly British Museum, Natural History (BMNH)) confirms that it is deposited in NHMUK, rather than in the Muséum National d’Histoire Naturelle, Paris (MNHN). The Titan database (Tavakilian and Chevillotte 2026) [9] and the Catalogue of Chinese Coleoptera (Lin and Tavakilian 2019: 339) [8] incorrectly list MNHN as the depository of the holotype. These records are corrected here.

***Dolophrades punctatus* (Pic, 1934) stat. restit.**

Chinese common name: 南投朵天牛

Figure 3

*Dorcadiohammus punctatus* Pic, 1934: 8 [3]. Type locality: China (Taiwan: Fainan).

*Dolophrades punctatus*: Breuning, 1961: 309 (catalogue) [11]; Hua 2002: 204 (catalogue) [12].

*Dolophrades annulicornis*: Gressitt, 1951: 329 (catalogue) [16]; Hua 2002: 204 (catalogue) [12]; Löbl & Smetana 2010: 289 (catalogue) [14]; Lin & Tavakilian 2019: 339 (catalogue) [8].

**Diagnosis.** *Dolophrades punctatus* (Pic, 1934) is similar to *D. subdenudatus* Gressitt, 1938, but differs in the following characters: elytra distinctly constricted immediately behind the humeri and then gradually widened towards the middle, whereas the lateral margins are nearly subparallel behind the humeri in *D. subdenudatus*; basal elytral punctures relatively sparse, whereas finer and denser in *D. subdenudatus*; antennae relatively stouter and shorter, whereas more slender and elongate in *D. subdenudatus* also described two indistinct grey fasciae on the elytra, whereas Gressitt (1938) described irregular glabrous patches on the elytra of *D. subdenudatus*.

**Redescription.** Male. Body length 9.36 mm, humeral width 2.6 mm.

Body reddish-brown to dark brown, with fine greyish-brown pubescence. Head densely punctate; frons wider than high, median longitudinal groove extending from anterior margin to occiput. Antennal tubercles raised, narrowly separated. Antennae very long and slender, about 2.4 times as long as body, testaceous, with antennomeres brownish apically; scape subcylindrical, with an apical cicatrix; antennomere 3 longest; antennomere 4 about 1.8 times as long as scape; antennomere 11 about 1.4 times as long as scape; bases of antennomeres 3–11 with indistinct greyish pubescence. Pronotum longer than wide, with transverse grooves near anterior and posterior margins; lateral tubercles short, robust, apically obtuse; disc uneven, densely punctate. Scutellum densely covered with pubescence. Elytra slightly wider than pronotum; humeri prominent; elytra distinctly constricted immediately behind humeri, slightly expanded towards middle, then gradually narrowed posteriorly; apices separately rounded; disc punctures similar in size to those on head and pronotum, partly arranged in irregular longitudinal rows and becoming finer towards apices. Ventral surface finely pubescent. Prosternum sparsely punctate; prosternal process narrow, lower than procoxae, gradually widening apically; procoxal cavities slightly open posteriorly. Mesosternal process without tubercle; mesocoxal cavities open laterally to mesepimera. Abdominal ventrite I longest, as long as ventrites II and III combined. Legs relatively slender, femora fusiform; metafemur not reaching apex of abdominal ventrite V; first metatarsomere as long as following two combined.

**Figure 3:**
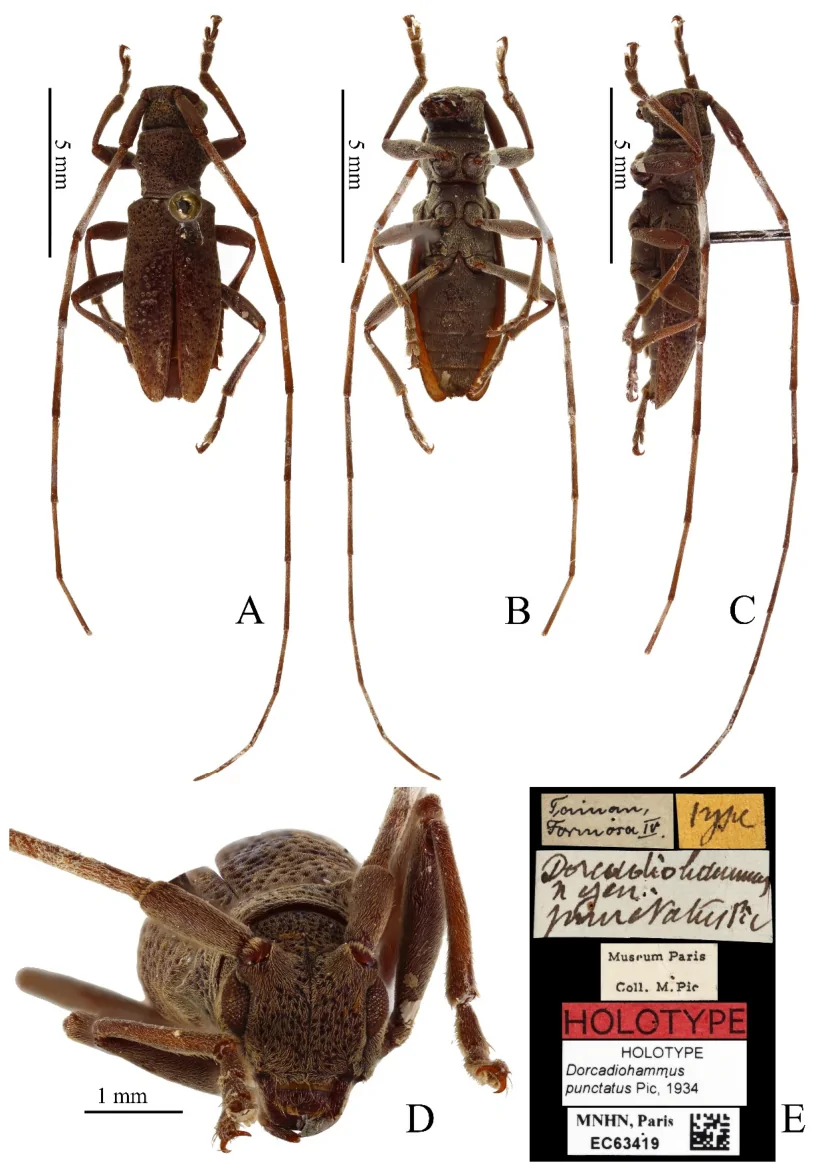
Holotype of *Dorcadiohammus punctatus* Pic, 1934. (**A**) Dorsal view; (**B**) ventral view; (**C**) lateral view; (**D**) frontal view; (**E**) labels.

**Type material examined.** Holotype of *Dorcadiohammus punctatus* Pic, 1934 male (MNHN, EC63419); label details are shown in Figure 3E.

**Distribution.** China (Taiwan: Fainan = Tainan).

**Comments.*** Dorcadiohammus punctatus* Pic, 1934 was subsequently placed in synonymy with the taxon treated as *Dolophrades annulicornis* (Schwarzer, 1925). For earlier references under *D. annulicornis*, see “Taxon excluded from *Dolophrades*”. Pic (1934) described *D. punctatus* as a 9 mm beetle with relatively short elytra bearing two indistinct grey fasciae; these fasciae are not clearly discernible in the available dorsal photograph of the type specimen. Examination of the type photograph, together with comparison with the type material of *D. subdenudatus*, reveals consistent differences in elytral shape, basal elytral punctation, antennal proportions, and elytral pubescence, as detailed in the Diagnosis above. These differences support the recognition of *D. punctatus* as a distinct species. It is therefore removed from synonymy with *annulicornis* and reinstated as a valid species (stat. restit.). The original description gives the type locality as “Ile Formose: Fainan”, whereas the original label of the holotype reads “Tainan, Formosa”. The former is evidently a typographical error for Tainan.

***Dolophrades subdenudatus*** **Gressitt, 1938 stat. restit.**

Chinese common name: 埔里朵天牛

Figure 4

*Dolophrades subdenudatus* Gressitt, 1938: 153 [4], pl. I, fig. 6. Type locality: China (Taiwan: Hori (Horisha); Lingafelter, Garzón-Moreno & Nearns 2013: 138, fig. 86a (holotype) [17]; Lingafelter & al. 2014: 327, fig. 163q (holotype) [18].

*Dolophrades punctatus* m. *subdenudatus* Breuning, 1961: 309 [11] (catalogue).

*Dolophrades annulicornis*: Gressitt, 1951: 329 (catalogue) [16]; Hua 2002: 204 (catalogue) [12]; Löbl & Smetana 2010: 289 (catalogue) [14]; Lin & Tavakilian 2019: 339 (catalogue) [8].

**Diagnosis.** *D. subdenudatus* is similar to *D. punctatus*, but differs in the following characters: elytral lateral margins nearly subparallel immediately behind the humeri, whereas the elytra are distinctly constricted behind the humeri and then gradually widened towards the middle in *D. punctatus*; basal elytral punctures finer and denser, whereas relatively sparse in *D. punctatus*; antennae more slender and elongate, whereas relatively stouter and shorter in *D. punctatus*. In addition, Gressitt (1938) described irregular glabrous patches on the elytra of *D. subdenudatus*, whereas Pic (1934) described two indistinct grey fasciae on the elytra of *D. punctatus*.

**Redescription.** Male. Body length 9.6 mm, humeral width 2.5 mm.

Body reddish-brown to dark brown, largely covered with yellowish-grey or yellowish-brown pubescence; elytra with irregular dark glabrous patches. Head densely punctate; frons wider than high, median longitudinal groove extending from anterior margin to occiput. Antennal tubercles prominent, narrowly separated. Antennae slender, about 2.5 times as long as body; scape subcylindrical, with cicatrix at apex; antennomere 3 longest; antennomere 4 about 2 times as long as scape; antennomere 11 about 1.8 times as long as scape; bases of antennomeres 3–11 indistinctly greyish pubescence. Pronotum longer than wide, weakly constricted near anterior and posterior margins; lateral tubercles short, robust, apically obtuse; disc uneven, densely punctate except below lateral tubercles. Scutellum densely covered with golden pubescence. Elytra elongate, humeri slightly prominent; lateral margins nearly subparallel immediately behind humeri, then gradually narrowed posteriorly; apices separately rounded; disc punctures coarser than those on head and pronotum, partly arranged in irregular longitudinal rows, becoming sparser posteriorly. Prosternum medially sparsely punctate; prosternal process narrow, lower than procoxae, gradually widening apically; procoxal cavities slightly open posteriorly. Mesosternal process without tubercle; mesocoxal cavities open laterally to mesepimera; mesepisternum bearing 3–4 sparse punctures. Abdominal ventrite I longest, as long as ventrites II and III combined. Legs long, femora fusiform; mesotibiae with an oblique groove near outer apex; metafemur not reaching apex of abdominal ventrite V; first metatarsomere as long as following two combined.

**Figure 4:**
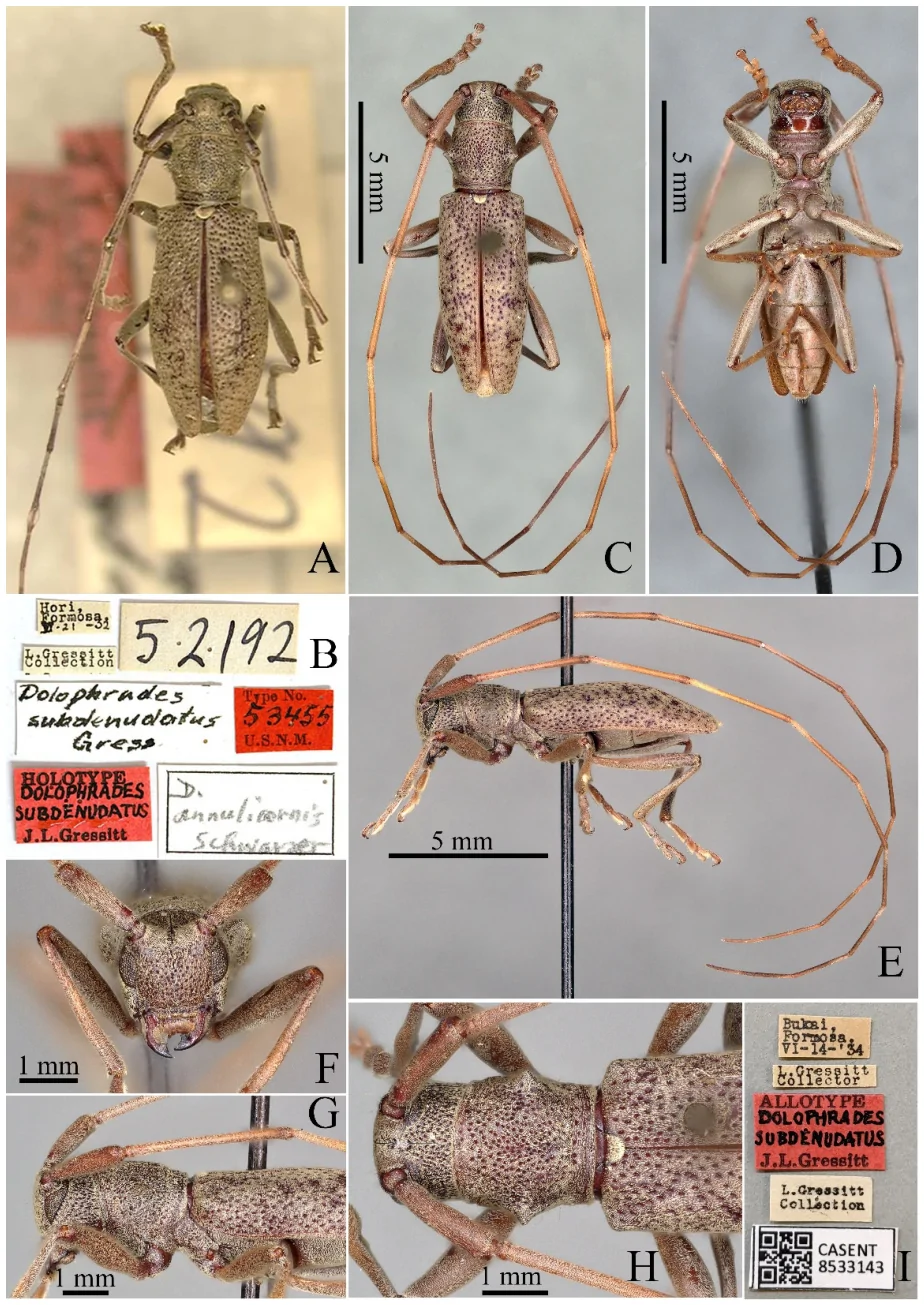
Holotype and paratype of *Dolophrades subdenudatus* Gressitt, 1938. (**A**,**B**) Holotype, female (USNM); (**C**–**I**) paratype, male, originally designated as “allotopotype” (CAS, CASENT8533143). (**A**,**C**) Dorsal view; (**D**) ventral view; (**E**) lateral view; (**F**) frontal view; (**G**) lateral view of pronotum; (**H**) dorsal view of pronotum; (**B**,**I**) labels.

**Type material examined.** Holotype of *Dolophrades subdenudatus* Gressitt, 1938, female (USNM; Figure 4A,B), from Hori (Horisha), Taiwan, 650 m, 21 June 1932, cited as no. 52192 in the original description and also bearing a USNM type label numbered 53455. Paratype, male (CAS, CASENT8533143; Figure 4C–I), originally designated as “allotopotype” by Gressitt (1938) and labelled “ALLOTYPE”, from Bukai, east of Hori, Taiwan, 900 m, 14 June 1934. Locality, elevation, and collection-date information are supplemented from the original description; specimen labels are illustrated in Figure 4B,I.

**Distribution.** China (Taiwan: Nantou County, Hori = Puli).

**Comments.** *Dolophrades subdenudatus* Gressitt, 1938 was subsequently treated as a junior synonym of the taxon then recognized as *D. annulicornis*, a treatment followed by later authors. For earlier references under *D. annulicornis*, see “Taxon excluded from *Dolophrades*”. Examination of the original description and photographs of the female holotype and male paratype of *D. subdenudatus*, together with comparison with the holotype of *D. punctatus*, reveals consistent differences in elytral shape, basal elytral punctation, antennal proportions, and elytral pubescence, as detailed in the Diagnosis above. In particular, the antennal comparison is based on the male paratype of *D. subdenudatus* and the male holotype of *D. punctatus*, thereby avoiding possible effects of sexual dimorphism. These differences support the recognition of *D. subdenudatus* as a distinct species. The taxon formerly treated as *D. annulicornis* is here excluded from *Dolophrades* and treated as *Peblephaeus decoloratus annulicornis* (Schwarzer, 1925), stat. restit. Consequently, *D. subdenudatus* is removed from synonymy with *annulicornis* and reinstated as a valid species (stat. restit.).

The specific epithet *subdenudatus* refers to the locally reduced or denuded elytral pubescence described by Gressitt (1938). However, this condition is not unique to the species, as uneven or locally reduced elytral pubescence producing a mottled appearance also occurs in other species of the genus, including *D. tianmushanensis* sp. nov. To avoid implying that this character is uniquely diagnostic, the Chinese common name “埔里朵天牛” is adopted here, referring to the type locality, Hori (Horisha; present-day Puli), Taiwan, China.

***Dolophrades birmanus*** **Breuning, 1958**

Chinese common name: 缅朵天牛

Figure 5

*Dolophrades birmanus* Breuning, 1958: 3 [6], fig. 1. Type locality: Myanmar (Birmanie: Ruby Mines (Mogok)); Breuning 1961: 309 (catalogue).

**Redescription.** Female. Body length 13 mm, humeral width 4 mm. Body dark brown, densely covered with silky brown pubescence. Head densely punctate; frons wider than high, median longitudinal groove extending from anterior margin to occiput. Lower eye lobes slightly longer than the genae. Antennal tubercles prominent, widely separated. Antennae slender, approximately one-third longer than the body (♀); scape robust, more strongly tapered towards base, with cicatrix at apex; antennomere 3 much longer than scape. Pronotum wider than long, lateral tubercle large, strong, apex acute; disc uneven, densely punctate except below lateral tubercles. Scutellum thickly covered with yellow pubescence. Elytra elongate-oval, humeri nearly straight; disc punctures coarser than those on head and pronotum. Mesepisternum without punctures. Legs moderately long, femora fusiform, metafemur not reaching apex of abdominal ventrite V; metatarsomere I longest, not longer than II+III combined.

**Figure 5:**
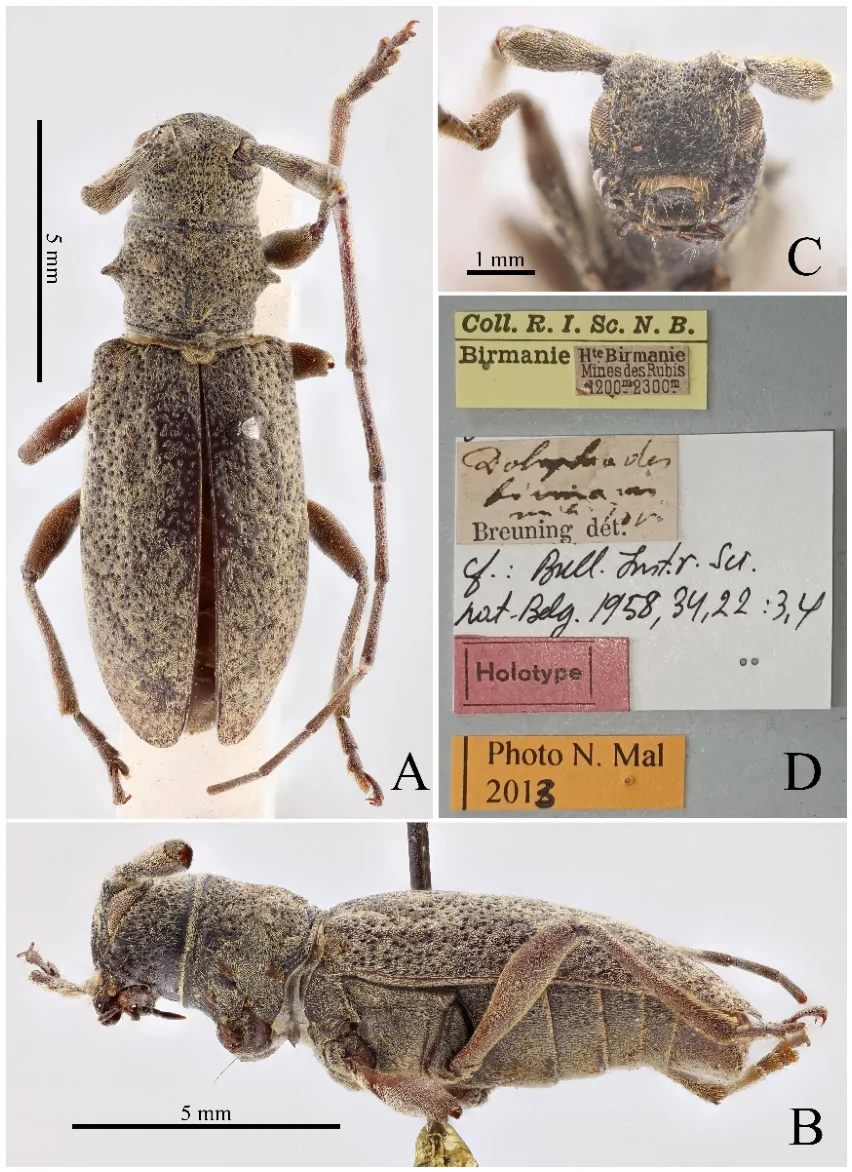
Holotype of *Dolophrades birmanus* Breuning, 1958. (**A**) Dorsal view; (**B**) lateral view; (**C**) frontal view; (**D**) labels.

**Type material examined.** Holotype of *Dolophrades birmanus*, female (IRSNB); label details are shown in Figure 5D.

**Distribution.** Myanmar (Mogok, Ruby Mines).

***Dolophrades mustanganus*** **Holzschuh, 2003**

Chinese common name: 玛坦朵天牛

*Dolophrades mustanganus* Holzschuh, 2003: 308 [7], pl. VI, fig. 8. Type locality: Nepal (Dhawalagiri, Kali-Gandaki-Khola, Kalopani); Weigel 2006: 502 (distribution) [19]; Löbl & Smetana 2010: 289 (catalogue) [14].

**Comments.** The original description of *Dolophrades mustanganus* includes a specimen photograph, but its quality is insufficient for reproduction here. As the type specimen is deposited in the private collection of the original author and was not available for re-examination, the morphological information and comparisons presented here are based on the original description and published figure of Holzschuh (2003). *D. mustanganus* is similar to *D. terrenus* Bates, 1884 in having the basal four antennomeres distinctly thickened in the male, but differs in having larger eyes, antennal tubercles less widely separated, antennomeres without pale basal annulations, and more uniformly coarse elytral punctation. It differs from *D. birmanus* Breuning, 1958 mainly in having smaller, more obtuse pronotal lateral tubercles and a less distinct apical cicatrix on the scape.

**Distribution.** Nepal (Dhawalagiri, Kali-Gandaki-Khola, Kalopani).

***Dolophrades tianmushanensis*** **Yang, Xie & W. Wang, sp. nov.**

http://zoobank.org/NomenclaturalActs/903b7133-7887-4028-9cb0-cdff34f0bb6c (accessed on 5 October 2026)

Chinese common name: 天目山朵天牛

**Figure 6:**
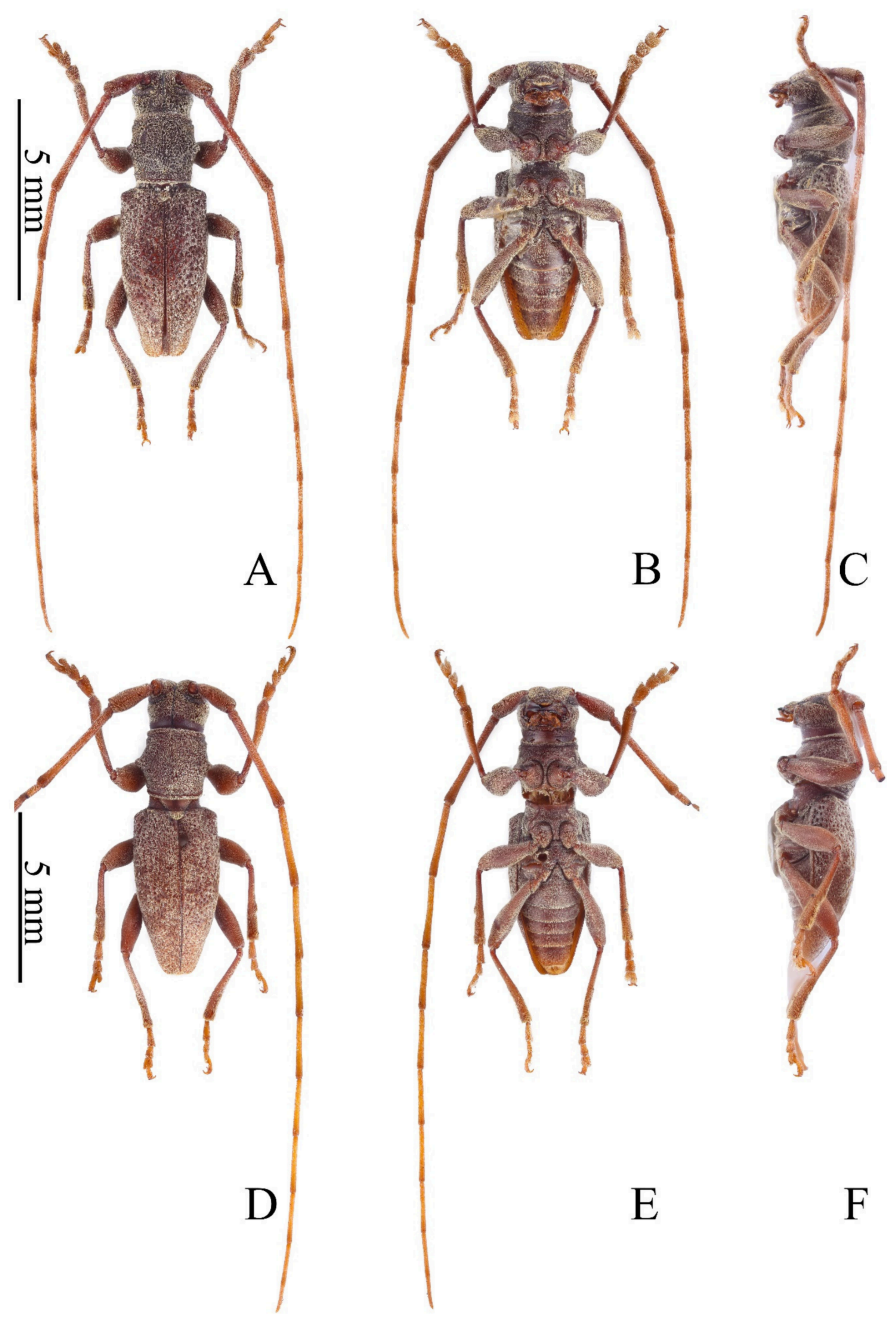
Habitus of *Dolophrades tianmushanensis* Yang, Xie & W. Wang, sp. nov. (**A**–**C**) Holotype male; (**D**–**F**) paratype male. (**A**,**D**) dorsal view; (**B**,**E**) ventral view; (**C**,**F**) lateral view.

**Figure 7:**
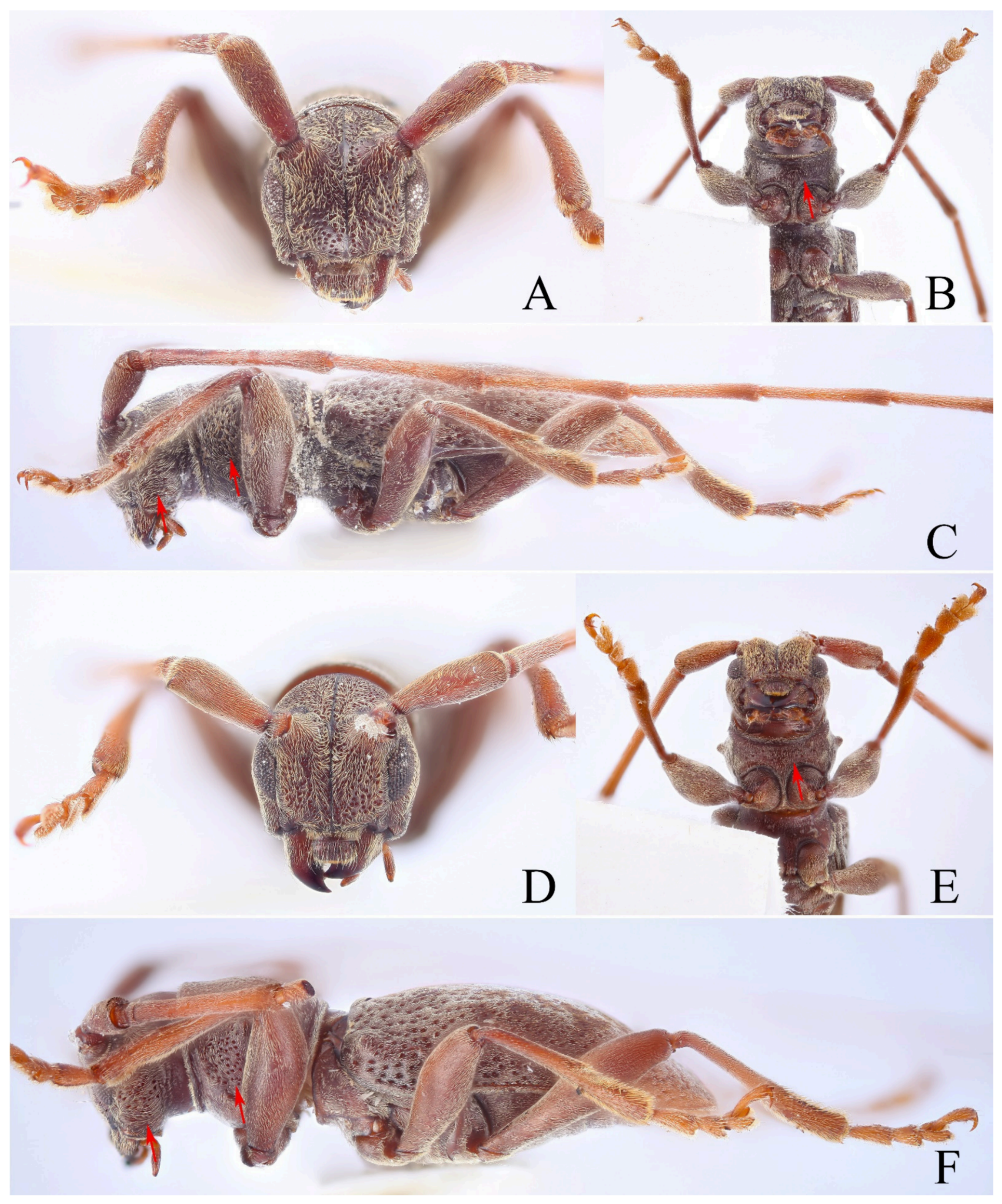
Habitus of *Dolophrades tianmushanensis* Yang, Xie & W. Wang, sp. nov. (**A**–**C**) Holotype male; (**D**–**F**) paratype male. (**A**,**D**) frontal view; (**B**,**E**) ventral view; (**C**,**F**) lateral view. Red arrows indicate the shallow coarse punctures on the prosternum (**B**,**E**), and the dense punctation below the pronotal lateral tubercles and on the genae (**C**,**F**).

**Figure 8:**
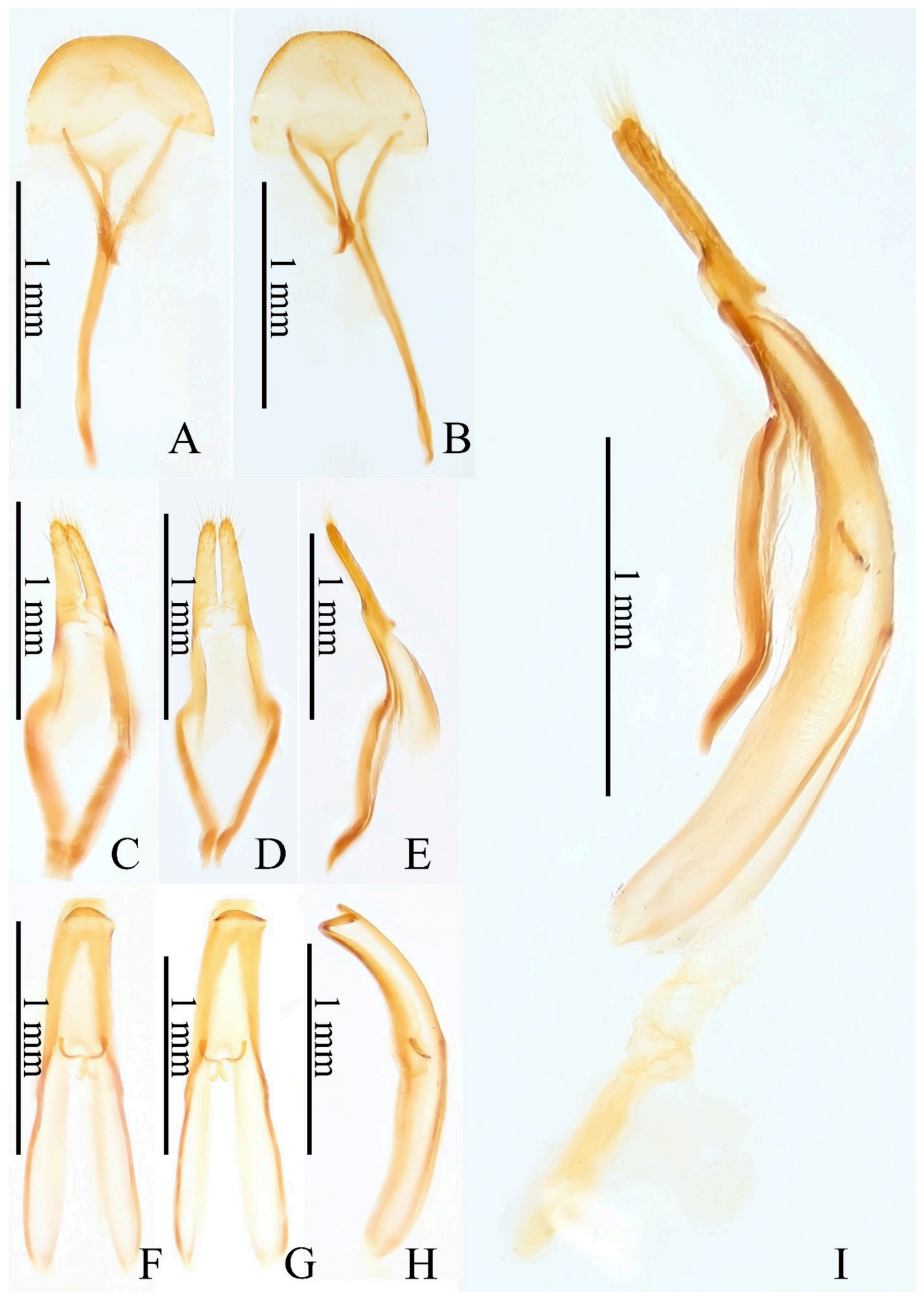
The genital structures of *Dolophrades tianmushanensis* Yang, Xie & W. Wang, sp. nov. (**A**,**B**) Abdominal segments VIII–IX; (**C**–**E**) tegmen; (**F**–**H**) median lobe; (**I**) male genitalia; (**A**,**C**,**F**) dorsal view; (**B**,**D**,**G**) ventral view; (**E**,**H**,**I**) lateral view.

Figure 6, Figure 7 and Figure 8

**Diagnosis.** This species is similar in general body shape to *D. subdenudatus*, but differs in having the scape robust and distinctly thickened in the apical half, whereas the scape is subcylindrical and relatively slender in *D. subdenudatus*; the elytral lateral margins are subparallel from the humeri to the middle and strongly constricted at the apical 1/3, whereas they are nearly subparallel behind the humeri and gradually narrowed posteriorly in *D. subdenudatus* (Figure 4C). The new species is also similar to *D. punctatus*, but differs in having the scape robust and distinctly thickened in the apical half, whereas it is subcylindrical and relatively slender in *D. punctatus* (Figure 3A); the elytral lateral margins are subparallel from the humeri to the middle and strongly constricted at the apical 1/3, whereas they are distinctly constricted immediately behind the humeri, slightly widened towards the middle, and gradually narrowed posteriorly in *D. punctatus*.

**Description.** Male (Figure 6A–F and Figure 7A–F). Body length 7.1–8.5 mm, maximum width across middle of elytra 2.2–2.6 mm, humeral width 2.1–2.4 mm; herewith the holotype is 7.1, 2.2 and 2.1 mm, respectively.

Body reddish-brown to blackish brown. Head blackish brown, densely covered with pubescence; maxillary and labial palps reddish-brown. Antenna light reddish-brown, apices of antennomeres 3–10 dull brown, densely covered with greyish-yellow pubescence. Legs mostly dull reddish-brown, tarsi light reddish-brown. Pronotum densely covered with pubescence, with a small, indistinct pubescent patch at base of middle line. Scutellum densely clothed with recumbent, greyish-yellow pubescence. Elytra pubescence unevenly distributed, partly denser and forming mottled maculae.

Head densely punctate, frons wider than high, median longitudinal groove extending from anterior margin to occiput; lower eye lobes longer than broad, distinctly shorter than genae in frontal view; vertex moderately arcuate. Antennal tubercles prominent, narrowly separated; antennae about 2.1 times as long as body; scape robust, distinctly thickened apically, with an apical cicatrix; pedicel knob-like, distinctly thinner than scape; antennomere 3 longest, 2.1 times as long as scape; antennomeres 4–10 successively decreasing in length, antennomere 11 slightly arcuate, tapering from the middle, 1.8 times as long as scape.

Pronotum longer than broad, posterior margin narrower than anterior margin; disc convex, densely punctate, including below lateral tubercles; lateral tubercles small, short and obtuse. Scutellum tongue-shaped, rounded apically.

Elytra oval, 1.9 times as long as humeral width, widest across middle; humeri prominently produced, forming small, distinct tubercles; humeral width 1.5 times width pronotal base. Lateral margins subparallel from humeri to middle, then strongly constricted at apical 1/3; apices rounded. Surface densely punctate, punctures larger than those on head and pronotum, gradually becoming finer towards apices.

Prosternum inconspicuously wrinkled, medially with several shallow, coarse punctures; prosternal process almost smooth, narrow, lower than procoxae, gradually widening apically; procoxal cavities slightly open posteriorly. Mesosternum, mesepisternum without punctures; mesosternal process slightly convex apically; mesocoxal cavities open laterally to mesepimera. Metasternum without coarse punctures; metanepisternum wedge-shaped, slightly tapering posteriorly, anterior margin nearly straight and oblique, ventral margin gently bisinuate. Abdomen ventrites smooth; apex of terminal abdominal ventrite emarginate.

Legs moderately long; femora fusiform, metafemora not reaching abdominal apex; tibiae approximately as long as femora. Metatarsomere I longest, but not longer than metatarsomeres II and III; metatarsomere III deeply bilobed.

**Male genitalia** (Figure 8A–I). Tergite VIII semicircular, about half as long as wide, apical margin with sparse short setae. Tegmen widest at middle, moderately curved ventrally in lateral view; parameres about 1/4 the length of tegmen, distinctly elongate, gradually converging toward rounded apices, apices close to each other; each paramere bearing short setae of various lengths and longer setae near apex; tegminal struts connected basally. Median lobe longer than tegmen, about 1.2 times as long as tegmen, distinctly curved ventrally in lateral view; dorsal plate truncate at apex; ventral plate longer than dorsal plate, spatulate at apical margin.

**Female.** Unknown.

**Type material examined.** Holotype: China: male (ICYZU), Zhejiang Province, Hangzhou City, Linan District, Xitiannmushan, Kaishanlaodian, July 13, 2012, Guanglin Xie leg. Paratypes: one male (ICYZU), same data as holotype; one male (ICYZU), China: Zhejiang Province, Hangzhou City, Linan District, Xitiannmushan Natural Reserve, Laodian, June 13, 2009, Guanglin Xie leg.; one male (ICYZU), China: Zhejiang Province, Hangzhou City, Linan District, Xitiannmushan, Xianrending, alt. 1,500 m, July 29, 2011, Guanglin Xie leg.

**Distribution.** China: (Zhejiang).

**Etymology.** The specific name is derived from the type locality, Tianmushan Mountain, Zhejiang Province, China.

Key to the species of the genus *Dolophrades* Bates, 1884

1. Scape cylindrical, relatively slender, weakly narrowed towards base . . . . . . . . . . . . . . . . . . . . . . . . . . . . . . . . . . . . . . 2
–  Scape robust, distinctly thickened, more strongly narrowed towards base . . . . . . . . . . . . . . . . . . . . . . . . . . . . . . . . . . 4
2. Humeri rounded, not distinctly produced . . . . . . . . . . . . . . . . . . . . . . . . . . . . . . . . . . . . . . . . . . . . . . . . ***D. terrenus* Bates**
–  Humeri distinctly produced . . . . . . . . . . . . . . . . . . . . . . . . . . . . . . . . . . . . . . . . . . . . . . . . . . . . . . . . . . . . . . . . . . . . . . . . . 3
3. Elytral lateral margins nearly subparallel immediately behind humeri; basal elytral punctures finer and denser; antennae relatively more slender and elongate . . . . . . . . . . . . . . .. . . . . . . . . . . . . . . . . . . . . . . . . . . . . . . . . . . . . . . . . . . . ***D. subdenudatus* Gressitt**
–  Elytra lateral margins distinctly constricted immediately behind humeri, then slightly widened towards middle and gradually narrowed posteriorly; basal elytral punctures relatively sparser; antennae relatively stouter . . . . . . . . . . . . . ***D. punctatus* (Pic)**
4. Basal four antennomeres distinctly thickened in male . . . . . . . . . . . . . . . . . . . . . . . . . . . . ***D. mustanganus* Holzschuh**
–  Basal antennomeres other than scape not distinctly thickened . . . . . . . . . . . . . . . . . . . . . . . . . . . . . . . . . . . . . . . . . . . . 5
5. Pronotum wider than long; lateral tubercles long and apically acute; elytral lateral margins evenly arcuate and gradually converging posteriorly . . . . . . . . . . . . . . . . . . . . . . . . . . . . . . . . . . . . . . . . . . . . . . . . . .. . . . . . . . . . . . . . . . . . . . ***D. birmanus* Breuning**
–  Pronotum longer than wide; lateral tubercles short and apically obtuse; elytral lateral margins subparallel from humeri to middle, then strongly constricted at apical 1/3 . . . . . . . . . . . . . . . . . . . . . . . . . . . . . . . . . . . . . . . . . . . . . . ***D. tianmushanensis* sp. nov.**

**Taxon excluded from *Dolophrades* Bates, 1884**

***Peblephaeus decoloratus annulicornis*** **(Schwarzer, 1925), stat. restit.**

Chinese common name: 灰角粗天牛

Figure 9

*Cereopsius decoloratus* var. *annulicornis* Schwarzer, 1925: 59 [2]. Type locality: China (Taiwan: Pilam).

*Blepephaeus decoloratus* m. *annulicornis* Breuning, 1961: 309 (catalogue) [11].

*Dolophrades annulicornis*: Gressitt, 1951: 329 (catalogue) [16]. Hua, 2002: 204 (catalogue) [12]; Löbl & Smetana 2010: 289 (catalogue) [14]; Lin & Tavakilian 2019: 339 (catalogue) [8].

**Figure 9:**
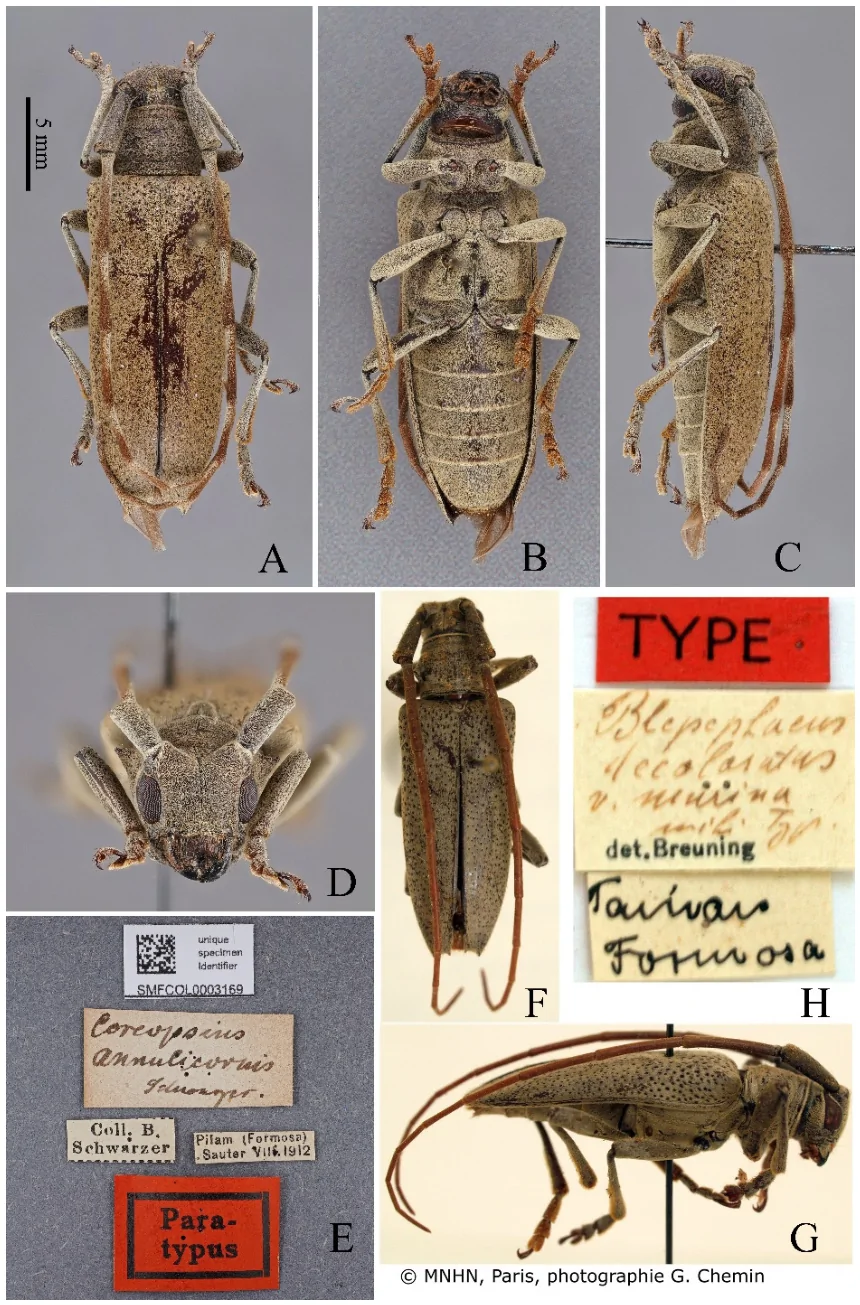
Type material of *Peblephaeus decoloratus* (Schwarzer, 1925) and *P. decoloratus annulicornis* (Schwarzer, 1925). (**A**–**E**) Paratype specimen of *Cereopsius decolorata* var. *annulicorne* Schwarzer, 1925, SMF; (**F**–**H**) type specimen of *Cereopsius decolorata* Schwarzer, 1925, MNHN. (**A**,**F**) Dorsal view; (**B**) ventral view; (**C**,**G**) lateral view; (**D**) frontal view; (**E**,**H**) labels.

**Type material examined.** Type-series material of *Cereopsius decolorata* Schwarzer, 1925 (= *Peblephaeus decoloratus*), male (MNHN); label details are shown in Figure 9H; One female of the original type series of *Cereopsius decolorata* var. *annulicorne* Schwarzer, 1925, labelled “Para-typus” (SMF, SMFCOL0003169), from Pilam, Taiwan; labels shown in Figure 9E.

**Distribution.** (Taiwan: Pilam [present-day Beinan, Taitung County]).

**Comments.** Schwarzer (1925) described *Cereopsius decolorata* from Taiwan, and in the same paper, proposed var. *annulicorne* based on two females from Pilam. The latter was distinguished from the nominotypical form principally by the broad, oblique grey pubescence at the bases of antennomeres 3–10. Subsequent authors treated *annulicornis* as a species of *Dolophrades*, and *Dorcadiohammus punctatus* Pic, 1934 and *Dolophrades subdenudatus* Gressitt, 1938 were later placed in synonymy with it.

Examination of the available type-series of *Peblephaeus decolorata* and *P. decolorata* var. *annulicorne* shows that the two taxa agree closely in general body form and in major structural characters, particularly the configuration of the pronotum and the general outline of the elytra. The principal difference between them is the broad greyish pubescence at the bases of antennomeres 3–10 in *annulicornis*, as originally noted by Schwarzer (1925). More importantly, *Dolophrades* is characterized by the absence of hind wings, whereas *Peblephaeus* possesses fully developed membranous hind wings. This represents a fundamental generic difference and clearly excludes *annulicornis* from *Dolophrades* and treated here as a subspecies of *Peblephaeus decoloratus*, under the name *Peblephaeus decoloratus annulicornis* (Schwarzer, 1925), stat. restit.

The removal of *annulicornis* from *Dolophrades* also necessitates reassessment of taxa formerly placed in synonymy with it. Examination of the type material and original descriptions supports the recognition of *Dolophrades punctatus* (Pic, 1934) and *D. subdenudatus* Gressitt, 1938 as separate valid species, and both are reinstated in the present study.

## Data Availability

The original contributions presented in this study are included in the article. Further inquiries can be directed to the corresponding authors.
